# Injecting botulinum toxin into the treatment of blepharospasm

**Published:** 2018

**Authors:** Marius Nicolae Popescu, Stella Ioana Popescu, Corina Cristina Cernat, Ana Maria Boariu, Edward Călin, Ana Vieru, Ovidiu Muşat

**Affiliations:** * Ophthalmology Department, “Dr. Carol Davila” Central University Military Emergency Hospital, Bucharest, Romania; Elias University Hospital, Bucharest, Romania

**Keywords:** botulinum toxin A, dystonia, neurotoxin

## Abstract

The present paper is an introduction to this category of therapy, the injection of botulinum toxin into blepharospasm and, at the same time, an attempt to familiarize our readers with this concept. Our activity in this field started in 2014; the favorable results we have obtained since then have made us write this article.

**Abbreviations:** BEB = Abnormal Benign Essential Blepharospasm, TXB = Botulinum Toxin

## Introduction

Allan Scott originally used botulinum toxin (TXB) in humans for the treatment of strabismus in 1977. In 1985, the same author published a study on the use of botulinum toxin in the treatment of blepharospasm [**1**,**2**].

In 1984, Frueh et al. have described the use of TXB A in blepharospasm. In the following years, injections of toxins became the first-line treatment for blepharospasm with a spectacular improvement resulting in more than 80% of the injected patients.

In 1985, Tsui et al. and in 1986 Brin et al. reported the results of open therapeutic trials with torticollis A toxin injections in patients who did not respond to other treatments and who were severely affected.

Between 1986 and 1991, starting with Tsui and continuing with Jankovic, Gelb, and Greene, at least five double blind, placebo-controlled studies focused on toxin A for cervical dystonia. All this culminated with an initial statement of indications for this drug at a consensus conference held at the National Institutes of Health (NIH) in the US in November 1990.

Currently, three TXB preparations, two TXB-A and one TXB-B are marketed; the rest of the serotypes are awaiting clinical trials to assess their role as therapeutic agents. The first two TXB-A, Botox® (Allergan, Irvine, USA) and Dysport® (Ipsen-Pharma, UK) commercial preparations have a long clinical experience in the first and less in the second. 

The third commercial preparation, NeuroBlock®/ Myoblock® (Elan Pharma, USA), is a TXB-B preparation approved for use in cervical dystonia with a reduced cumulative experience. A new BTX-A was recently introduced in China: Prosigne® (Lanzhou Institute of Biological Products, Shanghai, China). At present, TXB type A: Xeomeen (Merz Pharmaceuticals GmbH, Frankfurt, Germany) is available in a 100 U lyophilized bulb at the University Hospital Manuel Ascunce Domenech [**3**].

Botulinum toxin serotype A is one of the 8 neurotoxins produced by Clostridium botulinum gram-negative anaerobic bacillus. Its mechanism of action consists in blocking the action of acetylcholine in neuromuscular synaptic cleats. In this way, this leads to a temporary flaccid paralysis of the striated muscles.

Serotype B is an antigenically and mechanically distinct toxin produced by botulinum C, which also exerts its effects on the neuromuscular junction. Compared to type A toxins, Type B, which is used in a significantly different dose, has a faster onset and a higher diffusion in tissues; but the duration of the action is shorter. Patients treated with type B generally experience more inconvenience when injected and rates of satisfaction are generally lower.

**Fig. 1:**
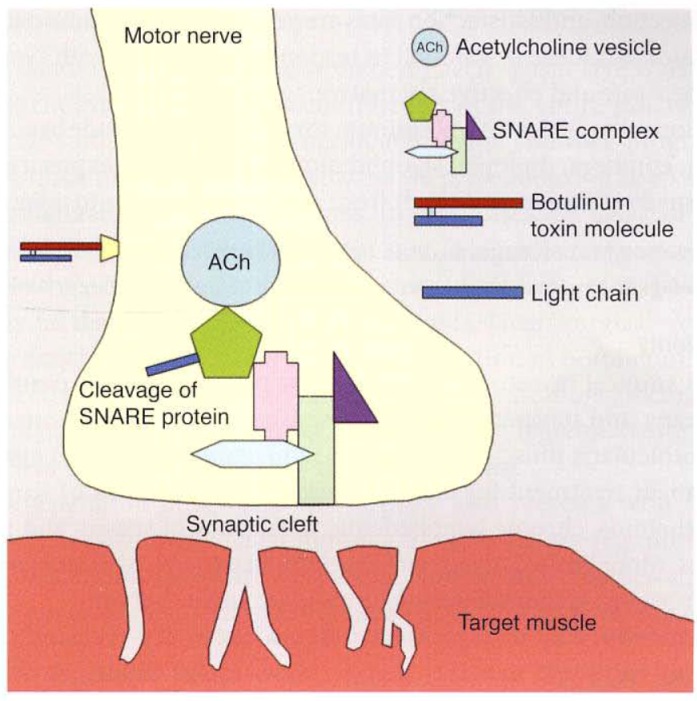
AAO - Orbit, Eyelids, and Lacrimal System, pp. 219 - Botulinum neurotoxin binds to specific membrane acceptors, allowing internalization of the light chain

## Definition

Benign Essential Blepharospasm (BEB) is a bilateral focal dystonia affecting about 30 out of 100,000 people [**4**].

In most cases, blepharospasm is an idiopathic disorder that usually lasts throughout life. Its severity may vary between frequent repeated blinking and persistent vigorous closure of the eyelids with functional blindness. In some cases, the coexistence of eye dryness syndrome can be observed.

A neurological examination eliminates systemic disease at the origin of this blepharospasm, such as Parkinson’s disease, supranuclear progressive paralysis, multiple systemic atrophy, or brain damage.

This disease comes in several forms, of varying severity. It can only affect one part (hemifacial spasm) or both sides. Blepharospasm can be isolated, affecting only the eyelids or accompanied by contractions of other facial muscles (Meige syndrome) or other parts of the body (generalized dystonia).

## Principles

In Spain, the product is sold in lyophilized form for therapeutic use (Botox®-Allergan) and for cosmetic use (Vistabel®-Allergan). Each vial contains 100 units of botulinum toxin. 

Before use, the lyophilized protein should be reconstituted with physiological saline (SF) without a preservative. The serum should be slowly poured into the vial to attenuate the vacuum and avoid shaking the container so as not to alter the stability of the protein molecule. The solution is prepared for a few minutes before application and it is used immediately. 

Since one vial contains 100 U botulinum toxin, we can say that, depending on the reconstitution, we will have more or less units per 0.1 cc. We usually work at 2.5 and 5.0 U. According to some authors, the efficacy of the prepared solution decreases by 44% after 12 hours after preparation.

The maximum dose of TXB per session is 400 U in adults. Higher doses (e.g., 500 U) favor neutralizing antibody production and resistance to treatment. The maximum recommended dose at the injection site is 50 U and the maximum site volume is 0.5 ml, except for special situations.

The minimum duration between two apps should be three to four months. Intervals less than this time have been shown to be a risk factor for developing resistance to TXB action. It will be applied at 4 months in the first 2 years and then every 6 months to avoid the development of antitoxin antibodies.

In France, three toxins are currently used: an average of 15 to 30 IU of Allergan toxin and 60 to 120 IU of toxin is injected into an eye. According to habits and symptoms, injections are made in pre-striatal (2 mm above the genes) or more distant in the orbicular muscle and under the forehead bone. 

Adding the corneal lubricant helps limiting the vicious circle (eye irritation by blinking) [**5**]. Good eye and eye hygiene and full ophthalmic examination can limit all local irritation factors (ametropia, dry eye, chronic conjunctivitis).

The presence of botulinum toxin in orbicular is checked by asking the patient to close his/ her eyelids very strongly. In the case of good impregnation, it is possible to open the patient’s eyelids with the fingers. This gesture is normally impossible.

The application technique consists in subcutaneous injection of TXB at the ends of both eyelids, with a thin needle, which should be laterally oriented to avoid the infiltration of the eyelid lifting muscle and to reduce the risk of poking. The same can happen with oculomotor muscles with transient diplopia. Occasionally, frontal and superciliary muscles may infiltrate. 

It should be clearly observed before application if the septal or palpebral part of the orbicular muscle is the one that makes the greatest contraction to define the ideal location of the muscle to receive the medicine. Note that the most common cause of treatment failure is an insufficient dose, which should be re-evaluated in each application.

For applications requiring higher doses, TXB can be applied in extra points, with a maximum of 10 U per point.

The application should be subcutaneous because the orbicular is located just beneath the skin, the palpebral, and the periocular area. It is performed in the operating room or in the consultation room with sterile material and an insulin syringe.

A complete assessment of the application schedule should be made before each session, as this may vary depending on the dose of muscle and muscles to be injected.

The difficulty of opening the eyelids when the orbicular does not forcefully contract may indicate that the patient suffers from a related but separate condition called apraxia of the eyelid opening.

In the apraxia of eyelid opening, the eyelids appear relaxed, and the eyebrows are often elevated as a result of the patient’s usual efforts to lift the upper eyelid. These two conditions (blepharospasm and apraxia of eyelid opening) may coexist and it is important to determine the contribution of apraxia eyelid opening in these patients as this condition does not respond well to injections of botulinum toxin.

## Complications

Eczema in the treated area, diplopia, infarction or overdose, influenza-like syndrome and antibody formation.

The most serious risk is the perforation of the globe at the time of injection in case of a sudden movement of the patient or, less likely, the poor assessment of anatomical plans. It is better to remain superficial even if it is less effective.

The angle of closure of glaucoma: botulinum toxin may cause angle closure glaucoma in people prone to it. You will be subject to strict medical supervision if necessary. If you have glaucoma, talk to your doctor about how this medicine can affect your medical condition [**6**]. 

Use of this contraindicated toxin during pregnancy, myasthenia gravis, Eaton-Lambert syndrome, and in hypersensitivity cases.

## Conclusions 

Toxin is the only etiological treatment for wrinkles of expression and blepharospasm. It is a safe medicine without significant side effects and if there are hypercorrections, they are transient.

However, remember that efficacy decreases after 4 months, is an expensive medicine and the molecule is not stable.

The art of botulinum toxin injections consists of correctly selecting muscles and setting the appropriate dose to reduce muscle spasticity. Sometimes an electromyograph is used to guide the injections. The botulinum toxin effect starts in 2-14 days and lasts for an average of 3-6 months.

Future studies should investigate technical factors such as optimal treatment intervals, different injection techniques, doses, types of botulinum toxins and formulations. Other issues are service delivery, quality of life, long-term efficacy, safety, and immunogenicity.
